# Post-infectious and non post-infectious irritable bowel syndrome: A comparative study

**DOI:** 10.12669/pjms.321.8628

**Published:** 2016

**Authors:** Jianbo Wang, Shihua Lu, Shijie Zhao

**Affiliations:** 1 Dr. Jianbo Wang, Department of Digestion Internal Medicine, The Affiliated Hospital of Shandong University of, Traditional Chinese medicine, Jinan, Shandong province, China; 2 Dr. Shihua Lu, Department of Medical Affairs, The Affiliated Hospital of Shandong University of, Traditional Chinese medicine, Jinan, Shandong province, China; 3 Dr. Shijie Zhao, Department of Digestion Internal Medicine, The Sixth People’s Hospital of Jinan, Jinan, Shandong province, China

**Keywords:** Post-infectious irritable bowel syndrome (PI-IBS), None post-infectious irritable bowel syndrome (NPI-IBS), Intestinal fatty acid binding protein (IFABP), Self-rating anxiety scale (SAS), Gastrointestinal symptom rating scale (GSRS)

## Abstract

**Objective:**

To compare the post-infectious irritable bowel syndrome (PI-IBS) and none post-infectious irritable bowel syndrome (NPI-IBS) clinically and experimentally.

**Methods:**

From May 2013 to January 2015, eighty-nine patients with irritable bowel syndrome (IBS)were recruited in the internal department of the affiliated hospital of Shandong University of Traditional Chinese Medicine. The clinical data were collected for all the patients, and a blood sample was collected to detect the level of C-reactive protein (CRP) and intestinal fatty acid binding protein (IFABP), an investigation questionnaire of gastrointestinal symptom rating scale (GSRS) and self-rating anxiety scale (SAS) were carried out to evaluate the gastrointestinal function and anxiety status.

**Results:**

In the study, forty-eight patients were included in PI-IBS group and 41 in Non-PI-IBS group. There was no significant difference in age, gender and GSRS between the two groups (p>0.05). In PI-IBS group 70.8% patients presented with the primary symptom of diarrhea and 60.4% presented with a SAS scores over 50, but in Non-PI-IBS group, the values were only 19% (p<0.05) and 34.1% (p<0.05). The level of IFABP and CRP were significantly higher in PI-IBS group than those in Non-PI-IBS group (p<0.05).

**Conclusion:**

The PI-IBS may be different from Non-PI-IBS in mechanism and should be treated using different strategies.

## INTRODUCTION

Irritable bowel syndrome (IBS) is a common intestinal disorder characterized by persistent or intermittent abdominal pain or discomfort, distention, and changes in stool patterns.^1^ It is reported that 3% to 30% of patients develop IBS after intestinal infection.^2^ Irritable bowel syndrome (IBS) which occured after an initial episode of acute gastrointestinal infection was defined as post-infectious irritable bowel syndrome (PI-IBS).^3^ Some studies suggest that low-grade inflammation plays an important role in the development of IBS^4^ and some authors have reported the high expression of blood level of cytokine such as TNF- α, IL-6, IL-8, IL-10 and IL-1β were found in patients with PI-IBS.^5^ In our opinion, the mechanism of PI-IBS may be different from non PI-IBS, resulting in some different manifestations between PI-IBS and non PI-IBS. However, few studies have been published on this topic in English literatures.

Intestinal fatty acid binding protein (IFABP) is a 15-kDa cytoplasmic protein located in small intestinal enterocytes involved in the uptake and transport of polar lipids such as fatty acids from the small-bowel lumen, which has been associated with injury to the intestinal mucosa and injury common to inflammatory bowel diseases.^6^ When the integrity of the enterocyte membrane is compromised, I-FABP are rapidly released into the circulation. This makes them a potentially suitable biomedical predictor of small bowel ischemia.^7^ Kittaka, in a clinical study of 37 patients diagnosed with small bowel obstruction, concluded the I-FABP level is a useful marker for discriminating between strangulated small bowel obstruction and simple small bowel obstruction.^7^ Using a prospective observational study of fifty patients with severe sepsis, Zhu found the IFABP concentrations in all patients were significantly increased.^8^ In addition, C-reactive protein (CRP) is an inflammation marker, confirmed by many studies. We speculate that as the PI-IBS is closely correlated with low-grade infection, the blood level of IFABP or CRP in PI-IBS patients may be higher than common IBS patients, but up till now, this viewpoint haven’t been confirmed.

Therefore, a comparative clinical study was carried out in the internal medicine department of our hospital. The objectives of this study was to compare the PI- IBS and none PI-IBS clinically and experimentally, to help physicians better identify and make treatment strategies for the disease.

## METHODS

From May 2013 to January 2015, Eighty-nine IBS patients were recruited in the internal medicine department of the affiliated hospital of Shandong university of Traditional Chinese medicine for this study, including 48 PI-IBS patients with a history of acute enteritis, bacillary dysentery or related gastrointestinal infection within the previous 3 to 12 months and 41 non-PI-IBS patients. The diagnosis of IBS was made as defined by the Rome III criteria. The patients with the following conditions were excluded: (1) gastrointestinal organic disease including peptic ulcer, Crohn’s disease, ulcerative colitis and pancreatitis; (2) history of major abdominal surgery; (3) evidence of cardiovascular, gastrointestinal, metabolic, psychological or malignant disease; and (4) pregnancy or lactating. Patients who were using medications that could alter gastrointestinal function two weeks prior to enrollment, as well as patients taking nonsteroidal anti-inflammatory drugs, steroids, or antibiotics were excluded from the current study.^9^ The study was approved by the institutional review board of our hospital. All subjects provided written informed consent at the beginning of the study.

In the study, the clinical data including age, gender and times of diarrhea were collected for all the patients in two groups. To evaluate the gastrointestinal function and anxiety state, an investigation questionnaire of Gastrointestinal Symptom Rating Scale (GSRS)^10^ and Self-Rating Anxiety Scale (SAS)^11^ were carried out for the included patients. The GSRS contains 15 items which were combined into 5 symptom clusters including reflux, abdominal pain, indigestion, diarrhea and constipation, and uses a 7-point Likert scale ranging from “no discomfort” to “very much discomfort”. A higher score of GSRS demonstrates a greater discomfort.^9^ The SAS contains 20 items which measure the subject’s anxiety levels. Each item includes 1 of 4 responses ranging from A = never to D = very often. Responses to positively phrased questions are scored as follows: A = 1, B = 2, C = 3 and D = 4. Responses to negatively phrased questions are scored as A = 4, B = 3, C = 2, and D = 1. An SAS standard score ≥ 50 indicates conscious anxiety. Lower SAS scores indicate milder anxiety.^12^ In addition, a blood sample was collected from all patients to detect the level of C-reactive protein (CRP) and intestinal fatty acid binding protein (IFABP).

The statistical analyses were carried out using SPSS 20.0 (SPSS Inc., Chicago, IL, United States). The measurement data were presented as mean ± SD. The difference in age, CRP, IFABP, GSRS and SAS were compared by the Student’s *t*-test. The assessment of categorical variables such as gender were evaluated by chi-squared test. A *P* value < 0.05 was considered as statistical significance.

## RESULTS

In the study, eighty-nine IBS patients were recruited, among which 48 were included in PI-IBS group and 41 in Non-PI-IBS group. In 89 patients, forty-two were female and forty-seven were male. In terms of the aetiology in PI-IBS group, thirteen patients were bacillary dysentery (27.1%), four were salmonella infection (8.3%), twenty were acute gastroenteritis (41.7%), and eleven were unknown aetiology (22.9%).

The clinical data of two groups are listed in Table-I. There was no significant difference in age, gender and GSRS between the two groups (p>0.05). In the PI-IBS group 70.8% patients presented with the primary symptom of diarrhea, but in the Non-PI-IBS group, only 19% patients presented with the primary symptom. There was significant difference between the two groups (p<0.05). Also, in terms of evaluation of anxiety status, the percentage of patients with SAS scores>50 in PI-IBS group was 60.4%, and in Non-PI- IBS group was 34.1%, the values of SAS was significantly higher in PI-IBS group than those in Non-PI-IBS group (p<0.05).

**Table-I:** The clinical data in PI-IBS and Non-PI-IBS groups.

	PI-IBS	Non-PI-IBS	p value
Case numbers(n)	48	41	-
Age(years)	37±9.7	39±8.6	0.91
Gender(male/female)	23/25	19/22	0.88
Gastrointestinal Symptom Rating Scale	29.6±12.9	25.7±14.1	0.73
Zung Self-Rating Anxiety Scale>50 scores (n, %)	29(60.4%)	14(34.1%)	0.01
Times of diarrhea (>3 times),(n, %)	34(70.8%)	19(46.3%)	0.02
Intestinal fatty acid binding protein(ug/L)	40.8±16.9	18.9±13.1	0.02
C-reactive protein(mg/L)	9.57±3.58	4.39±1.47	0.03

**Fig.1:**
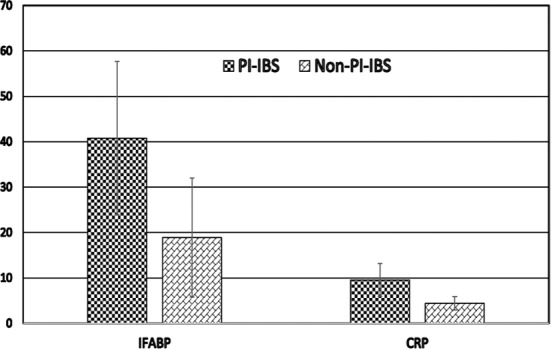
The comparison of intestinal fatty acid binding protein (IFABP, ug/L) and C-reactive protein (CRP, mg/L) in the two groups.

At the same time, the results of intestinal fatty acid binding protein and C-reactive protein in the two groups are shown in Table-I and Fig.1. It shows that both parameters were significantly higher in PI-IBS group than those in Non-PI-IBS group (p<0.05).

## DISCUSSION

Many authors have suggested that previous gastrointestinal infection or inflammation plays an important role in the pathogenesis of IBS.^3^,^13^ The high expression of TNF- α, IL-6, IL-8, IL-10, IL-1β and TGF-1β are significantly increased in PI-IBS patients, demonstrating the close association between infection and PI-IBS.^14^,^15^ In the current study, the CRP level in the PI-IBS group was significantly higher than that in the non PI-IBS group, which indicates the close correlation between gastrointestinal infection or inflammation and the development of PI-IBS, and confirms the viewpoints of low-grade infection.

At the same time, we found the percentage of patients with higher anxiety status in PI-IBS group were significantly higher than that in Non-PI-IBS group, demonstrating higher anxiety status is closely associated with the development of PI-IBS. In a study of 49 participants, Spence using logistic regressions found those who developed IBS had significantly higher levels of perceived stress, anxiety, somatisation and negative illness beliefs at the time of infection than those who did not develop IBS.^16^ Nicholl also suggested high levels of illness behavior, anxiety, sleep problems and somatic symptoms were found to be independent predictors of IBS.^17^ The current study drew the similar conclusion as the two above mentioned studies. The low grade inflammation of PI-IBS may result in the persistent symptoms, which adversely aggravate the negative emotion and cause the high anxiety status in patients with PI-IBS.

In addition, we found the level of IFABP in PI-IBS group was significantly higher than that in the Non-PI-IBS group, which confirmed our speculation before the study and indicated that the injury of intestinal mucosa is possible in PI-IBS. Therefore, we suggest that the PI-IBS may be different from Non-PI-IBS in the mechanism. Kittaka^7^ found the I-FABP level is elevated in strangulated small bowel obstruction and Wiercinska-Drapalo suggested a high level of I-FABP in ulcerative colitis,^18^ the two authors reported the similar viewpoints. Subsequently, we concluded from the current study that the treatment strategies of PI-IBS should be different from Non-PI-IBS.

However, our study has its limitations. First, the sample size was small, and from a large scale clinical study we may obtain more information. Second, from the study we suggest that the PI-IBS may have different mechanism when compared with Non-PI-IBS, but we didn’t perform further clinical or experimental study, and it remain unclear in the current study. Thus, more studies need to be carried out in the future.
